# Co-stimulators CD40-CD40L, a potential immune-therapy target for atherosclerosis: A review

**DOI:** 10.1097/MD.0000000000037718

**Published:** 2024-04-05

**Authors:** Simeng Tian, Yufei Wang, Jie Wan, Mao Yang, Zhenkun Fu

**Affiliations:** a Department of Immunology, Basic Medicine College, Heilongjiang Provincial Key Laboratory for Infection and Immunity, Harbin Medical University, Heilongjiang Academy of Medical Science, Harbin, China; b The First Affiliated Hospital of Harbin Medical University, Harbin, China; c Department of Neurosurgery & Nursing Teaching and Research Office, The Fourth Affiliated Hospital of Harbin Medical University, Harbin, China; d Department of Cardiology, The Fourth Affiliated Hospital of Harbin Medical University, Harbin, China.

**Keywords:** atherosclerosis, CD40, CD40 ligand, target-therapy

## Abstract

The interaction between CD40 and CD40 ligand (CD40L) a crucial co-stimulatory signal for activating adaptive immune cells, has a noteworthy role in atherosclerosis. It is well-known that atherosclerosis is linked to immune inflammation in blood vessels. In atherosclerotic lesions, there is a multitude of proinflammatory cytokines, adhesion molecules, and collagen, as well as smooth muscle cells, macrophages, and T lymphocytes, particularly the binding of CD40 and CD40L. Therefore, research on inhibiting the CD40-CD40L system to prevent atherosclerosis has been ongoing for more than 30 years. However, it’s essential to note that long-term direct suppression of CD40 or CD40L could potentially result in immunosuppression, emphasizing the critical role of the CD40-CD40L system in atherosclerosis. Thus, specifically targeting the CD40-CD40L interaction on particular cell types or their downstream signaling pathways may be a robust strategy for mitigating atherosclerosis, reducing potential side effects. This review aims to summarize the potential utility of the CD40-CD40L system as a viable therapeutic target for atherosclerosis.

## 1. Introduction

As a significant co-stimulatory and immune checkpoint pair, CD40 and CD40 ligand (CD40L, CD154) play a pivotal role in the adaptive immune response. CD40 and CD40L belong to the tumor necrosis factor receptor and its ligand superfamily members, and their interaction can induce immune response activation. Researches over the past 30 years have shown that the CD40-CD40L system plays a significant role in inflammatory or autoimmune diseases,^[1,2]^ notably including atherosclerosis.^[3,4]^ Of course, the previous research conducted in the clinical and basic fields on CD40-CD40L system remains controversial, making it difficult to determine its biological function. Atherosclerosis, characterized by the narrowing of arteries due to plaque buildup, linked to serious cardiovascular conditions like, including myocardial infarction, heart failure, and stroke. Recent studies have indicated that atherosclerosis can be viewed as a chronic inflammatory disease within the arterial walls where both the innate and adaptive immune systems play significant roles in its pathogenesis.^[5,6]^ The interaction between antigen-presenting cells (APCs) and T cells is crucial for the progression of vascular inflammation, with the co-stimulatory molecular family being key participants in APC-T cell interactions. This family has been implicated in numerous immune-related diseases for decades.^[7,8]^ In addition to the B7-CD28 members, CD40 and CD40L are also notable co-stimulatory molecules that catalyze the adaptive immune response. Hence, this review will discuss the immunological mechanisms involved in atherosclerosis formation and examine the compelling evidence supporting the involvement of CD40-CD40L in the development of atherosclerosis within blood vessels. Lastly, we’ll consider therapeutic strategies involving CD40-CD40L modifications in treatment of atherosclerosis.

## 2. The biological function of CD40-CD40L interaction

The binding of CD40 and CD40L, expressed on the surface of immune cells, can trigger signal transmission for functionality.^[9]^ It is recognized that the interaction between CD40L on T cells and CD40 on B cells (or APCs) is necessary for B cell differentiation and proliferation. Although activation of T and B cells response can process without the CD40-CD40L combination, many immune cellular biological functions show noticeable deficiencies, highlighting the vital roles of CD40-CD40L interaction, such as generation disorder of B cells and follicular helper T cells (TFH) in germinal center, or deficiencies of antibody class switching in B cells.^[10]^ Remarkably, alongside its regulation on B cells, the CD40-CD40L engagement significantly impacts hematopoietic or nonhematopoietic stem cells too.

On initiating a thymus-dependent humoral immune response, the connection between CD40 on B cells and CD40L on activated CD4 + T cells (helper T cell [Th]) plays a crucial role. The strength and duration of this receptor-ligand interaction are significantly important.^[11,12]^ The activation of B cells through the secondary costimulatory molecules-CD40-CD40L pathway can lead to the promotion of germinal center formation in peripheral immune organs, as well as the switching of immunoglobulin isotypes, somatic hypermutation, and terminal differentiation of B cells into plasma cells and memory B cells. Additionally, this signaling pathway can even contribute to the survival of B cells.^[13,14]^ Dendritic cells (DCs) play a critical role in promoting cytokine production, expressing co-stimulatory molecules, and facilitating the differentiation of Th cells through engagement with CD40 and CD40L on their surface when interacting with T cells. During the primary thymus-dependent antigen response, Th cells rapidly express CD40L and co-localize with plasmablasts in peripheral immune organs.^[15]^ Contrary to expectations, the CD40/CD40L pathway does not have any impact on the thymus-independent antigen response. Instead, within the germinal center, a specific type of activating T cell known as TFH plays a crucial role in the differentiation of B cells. The interaction between CD40L on TFH cells and CD40 on B cells regulates the activation of these 2 cell types. This binding promotes co-stimulatory over-expression and cytokine secretion, ultimately resulting in the proliferation and differentiation of B cells.^[16]^

In addition to the role in B cell-mediated humoral immunity, the CD40-CD40L pathway can also contribute to and play a crucial role in cell-mediated immunity, particularly in the progression of T cell-dependent autoimmune diseases.^[17,18]^ Interaction between CD40 and CD40L can induce the overexpression of CD80/86 in mature dendritic cells and promote the activation of T cell subgroups involved in graft rejection.^[19]^ Furthermore, blocking either CD40 or CD40L can have significant effects on T cell-mediated immunity, including T cell apoptosis and the development of regulatory T cells.^[20]^

As a vital member of the APC family, macrophages can express CD40 to modulate their immunological functions. Activating CD40 on macrophages results in the production of proinflammatory cytokines, increased expression of MHC and co-stimulatory molecules, as well as the release of nitric oxide and matrix metalloproteinases.^[21]^ Interestingly, the M2 phenotype and CD40 expression exhibit reciprocal inhibition, while the M1 phenotype displays the opposite pattern.^[22]^ In the context of the endovascular wall, the interaction between CD40 and CD40L expressed on endothelial cells and neutrophils promotes mutual adhesion.^[23]^ Therefore, it seems that the CD40-CD40L axis may contribute to the development and progression of vascular endothelial diseases, including atherosclerosis, with high probability.

## 3. CD40-CD40L signaling pathway

The interaction between CD40 and CD40L on the surfaces of various cell types can initiate distinct signal transduction pathways, serving specific functions individually. CD40L, expressed on T cell surfaces, plays a pivotal role in mediating 3 signaling pathways that regulate the activation, survival, proliferation of immune cells, as well as T cell responses. These pathways include the p38MAPK, extracellular regulated protein kinase (ERK), and PI3K signaling pathways, with particular emphasis on the p38MAPK pathway. CD40L triggers the expression of significant marker molecules and the secretion of functional cytokines through both the p38MAPK and ERK pathways, notably interleukin-12 (IL-12).^[24]^ In addition to these functions, CD40L also contributes to an antiapoptotic mechanism through these 3 signal pathways, primarily via the PI3K pathway.^[25]^

The CD40 molecule lacks a tyrosine kinase and protein kinase aggregation region due to its short intracellular segment. As a result, intracellular signal transduction mediated by CD40 necessitates the involvement of adaptor molecules, specifically members of the TNF receptor-associated factor (TRAF) family. To initiate downstream signal transduction and regulate cellular functions, CD40 must bind to TRAF family members through its cytoplasmic segment, characterized by the PXQX (T/S) site.^[26]^ Notable members of the TRAF family confirmed to participate in CD40 signal transduction include TRAF1, TRAF2, TRAF3, TRAF4, TRAF5, and TRAF6.^[14,27–29]^ The recruited TRAF molecules play a pivotal role in initiating downstream signaling cascades that give rise to various biological effects. These effects encompass anti-apoptotic responses, the activation and nuclear translocation of NF-κB, as well as the stimulation of ERK or JNK (c-Jun N-terminal kinase) pathways.^[27,30]^ Nevertheless, it’s worth noting that the TRAF family of proteins exhibits the bi-functional behavior, with their roles manifesting as either positive or negative regulators depending on the specific cell types involved. Furthermore, aside from binding to the intracellular segment of the CD40 molecule, TRAF family members also possess the ability to competitively interact with the intracellular segments of other co-stimulatory molecules, thereby eliciting diverse biological outcomes.^[26]^ In the initial stages of CD40 signal transduction, the tyrosine protein kinase belonging to the Src family comes into play by instigating downstream signaling events through auto-phosphorylation mechanisms. Among the key participants of the Src family in the intracellular CD40 signal transduction process, Lyn and Syk are of paramount importance. Lyn, residing in the cell membrane, auto-phosphorylates itself and subsequently recruits and activates Syk to transmit signals downstream.^[31]^

In addition to TRAF and the Src family, CD40 signal transduction is intricately linked to the mitogen-activated protein kinase (MAPK), NF-κB, and nuclear factor of activated T cells pathways.^[32,33]^ The MAPK family, comprising 3 primary members—ERK, JNK, and p38 MAPK, plays a vital role in the signaling transduction processes across various cell types. These kinases can mediate a range of biological effects, including cellular growth, proliferation, cytokine secretion, survival, and programmed cell death by activating transcription factors. In the context of CD40-mediated cholangiocyte apoptosis, it is noteworthy that this process hinges on the phosphorylation of STAT3, along with the sustained activation of JNK1/2 and ERK1/2.^[34]^ The engagement of CD40 also elicits transcriptional responses, such as the upregulation of anti-apoptotic genes like Bcl-2 and Bcl-X. This outcome is attributed to the enduring activation of ERK1/2 and its upstream regulator, MEK1/2, as a result of CD40 signaling.^[35]^ In the case of B cells, CD40 ligation can lead to the production of reactive oxygen species through the involvement of 5-lipoxygenase, ultimately culminating in the activation of p38 MAPK.^[36]^ Downstream molecules of CD40 signaling, such as TRAF1/2 and P38, can activate the NF-κB pathway, which further activates I-κK to catalyze I-κB phosphorylation, resulting in nuclear translocation of NF-κB. Exposure of nuclear localization signals in DNA binding sequences exerts a nuclear transcription effect to stimulate the up-regulation of some co-stimulatory molecules and cytokines, such as IL-6, IL-12, and TNF-α. CD40 signaling can also induce the mRNA transcription of NF-κB family member Rel-B to expand and sustain CD40 signaling.^[37]^ CD40 can trigger 2 distinct NF-κB activation pathways, resulting in the activation of NF-κB1 and NF-κB2. For B cells, CD40 signaling strongly activates NF-κB1 by NIK and TRAF3 to promote the phosphorylation of IKKα, but NF-κB2 regulation is indicated by TRAF3-independent mechanisms. So far, for other kinds of cells, both NF-κB1 and NF-κB2 pathways are activated by CD40 in T cells and DCs, and nuclear translocation of NF-κB binding activity is induced by CD40 in mono-/macrophages types through NF-κB1 activation.^[38]^

## 4. CD40-CD40L and atherosclerosis

Over the past 3 decades, numerous researches have highlighted the correlation between CD40/CD40L interactions and the occurrence and progression of atherosclerosis. Atherosclerosis is a chronic inflammatory disease that affects the walls of arteries, leading to plaque buildup and subsequent narrowing of the arteries. This can result in serious complications such as myocardial infarction, heart failure, stroke, and more. Risk factors for atherosclerosis include high cholesterol levels, high blood pressure, diabetes, smoking, obesity, family history, and both innate and adaptive immune systems play a role in its pathogenesis.^[39,40]^ During the early stages of atherosclerosis, T cells and monocytes adhere to the damaged vascular bed lining and transmigrate into the sub-endothelial space. As atherosclerosis progresses, monocytes differentiate into macrophages that engulf trapped lipoprotein particles and eventually transform into foam cells. In the meantime, chemokines released by these macrophages can further recruit monocytes, neutrophils, and lymphocytes from circulation. The foam cells may undergo apoptosis due to inadequate lipid clearance. However, if these cells are not cleared effectively, they may undergo necrosis and form the necrotic core of atherosclerosis.^[41,42]^ DCs have the ability to take up lipids and contribute to the formation of foam cells.^[43]^ Both macrophages, which are derived from monocytes, and DCs are classical APCs that play a crucial role in activating and shaping T cell responses. Co-stimulatory molecules, such as CD40-CD40L, serve as important signals for adaptive immune responses and also play significant roles in the development and progression of atherosclerosis. Overexpressing CD40 on low-grade inflammatory monocytes is a critical contributor to the pathogenesis of atherosclerosis. Activation of TRAM-mediated signaling processes in these monocytes leads to the activation of MAPK and STAT5, which ultimately result in the upregulation of CD40 expression.^[44]^

As we know, atherosclerosis is a chronic inflammatory disorder that progresses slowly. Numerous studies have demonstrated that both CD40 and CD40L are expressed on various types of cells in atherosclerotic lesions, including immune cells and nonimmune cells.^[45,46]^ Furthermore, the CD40-CD40L interaction has been found to induce the degradation of the extracellular matrix and the formation of necrotic cores within atherosclerotic plaques, leading to their rupture and subsequent cardiovascular events.^[47,48]^ In early animal model studies of the CD40-CD40L interaction in atherosclerosis, CD40L^−/−^ApoE^−/−^ mice showed a significant reduction in atherosclerotic plaque area with a stable phenotype characterized by collagen-rich, lipid-poor, and low inflammation (lacking macrophages and T cells). Anti-CD40L inhibition treatment in LDR^−/−^ mice also produced similar results.^[49]^ However, CD40L^−/−^ bone marrow-derived cell transplantation did not significantly improve atherosclerosis lesions.^[50]^ The expression of CD40 on adipocytes and endothelial cells of mice plays a crucial role in chronic inflammatory diseases. CD40 deficiency is associated with the promotion of structural features that are characteristic of a stable plaque phenotype, as well as a decrease in leukocyte adhesion to inflammatory cell migration and consecutive plaque formation in atherosclerosis. In addition, CD40 deficiency has been shown to reduce the burden of atherosclerosis.^[51,52]^ These findings suggest that decades of research on the CD40-CD40L interaction and atherosclerosis have identified a promising immune-therapy target for this disease with high probability.

Various types of immune cells infiltrate the atherosclerotic parenchyma, and a large number of these cells express CD40 or/and CD40L to play similar or different biological functions. Therefore, CD40-CD40L presents cell-specific effects in atherosclerosis. The activation of T cell-mediated immune responses requires 2 signals: HLA-antigen-TCR and co-stimulatory molecules. Interaction between CD40 on APCs and CD40L on T cells, which is one of the important second co-stimulatory signals, positively regulates T cell responses. As a chronic inflammatory disease, atherosclerosis is associated with and infiltrated by various types of inflammatory CD4 + T cells, particularly Ths. The proinflammatory Th1 response can upregulate atherosclerosis through the secretion of IFN-γ, TNF-α, IL-1, IL-6, and IL-12. On the other hand, Th2 response showed an antiatherogenic trend by producing IL4, IL-5, and IL-10.^[53]^ Regulatory T cells (Tregs), which are capable of producing inhibitory cytokines such as IL-10 and TGF-β, have been identified in the development of atherosclerosis and have been shown to play a role in inhibiting progression through modulation of lipoprotein metabolism.^[54]^ B cells, another kind of important adaptive immune cells, are relatively uncommon in atherosclerotic lesions. However, they may have an atheroprotective function in established lesions by acting on adjacent adventitia. The exact role of CD40 signaling on B cells in atherosclerosis remains unclear, but it is possible that oxLDL-specific IgM antibodies from B cells may be relevant to the development of atherosclerosis.^[46,55]^

In addition to adaptive immune cells, innate immune cells such as dendritic cells (DCs), neutrophils, and monocytes/macrophages also play a role in the occurrence and development of atherosclerosis lesions. However, there is limited evidence to suggest that DCs expressing CD40/CD40L are related to atherosclerosis. It has been demonstrated that DCs stimulated with constant CD40L can provide long-lasting IL-12 responses for Th1 response activation. Moreover, CD40 activation in DCs can impair lipid uptake, leading to attenuated atherosclerosis.^[7,56]^ Neutrophils, as an essential type of inflammatory immune cells, have been also observed in atherosclerosis lesions. Neutrophils can interact with platelets through CD40-CD40L interactions. Activated platelets restimulate neutrophils by releasing soluble CD40L (sCD40L). When sCD40L combines with CD40 on neutrophils, it releases reactive oxygen species, which stimulates more platelets.^[57,58]^ Macrophages accumulation within the vascular wall is a key feature of atherosclerosis. Research on atherosclerotic plaques shows that the proportion and number of macrophages within a plaque may be an indicator of plaque phenotype and stability.^[59]^ Ligation of CD40 on monocytes/macrophages can induce IL-12 secretion to stimulate CD40L expression on T cells and abundant IL-1β expression in the atherosclerotic lesion. In addition to these 2 proinflammatory factors, after CD40 ligation on macrophages and binding with CD40L on T cells, activated macrophages can synthesize and secrete matrix metalloproteinases, MIP-1, MCP-1, IL-8, TNF-α, IFN-γ, as well as tissue factor to affect thrombosis.^[60]^ Recent research has shown that the application of small molecule inhibitors that block the interaction between CD40 and TRAF6 can preserve CD40-mediated immunity by allowing CD40-TRAF2/3/5 interactions to be used for the treatment of atherosclerosis.^[61,62]^

In atherosclerotic lesions with activated vascular endothelium, there are also other components expressing CD40/CD40L besides immune cells, such as vascular smooth muscle cells, endothelial cells, and platelets.^[33,52,63]^ The surface expression of both CD40 and CD40L on these cells can lead to similar biological functions as that of monocytes/macrophages in atherosclerotic lesions, including the secretion of proinflammatory cytokines, adhesion, and co-stimulatory molecules expression, and the production of prothrombotic tissue factors.^[64,65]^ For vascular smooth muscle cells, ligation of CD40 can induce the production of metalloproteinases to promote plaque rupture,^[66]^ and CD40 signaling can activate the Src family kinase pathway, leading to the induction of MAPK activities.^[33,67]^

Platelets are one kind of important component in the circulatory system and play a crucial role in the inflammatory response and atherosclerotic plaque formation.^[68,69]^ In addition to T cells, platelets are the primary source of CD40L in both the circulation and tissues. Upon activation, platelets release clotting proteins, proinflammatory cytokines, and chemokines that contribute to the recruitment of monocytes to atherosclerotic arteries and accelerate plaque formation.^[33]^ The interaction between CD40L on platelets and CD40 on endothelial cells can trigger various inflammatory responses, including the expression of adhesion molecules and inflammatory cytokines, such as ICAM-1, MCP-1, IL-6, IL-8, and matrix metalloproteinases.^[52,70,71]^ During endothelium-platelet-monocyte interactions in the early stage of atherosclerosis, endothelial CD40 expression plays a crucial role in determining the atherosclerotic susceptibility sites.^[72]^ Since most of the circulating CD40L is expressed on platelets, they may play a significant role in the pathogenesis of atherosclerosis. CD40L is presented from the cytoplasm to the surface after platelet activation and can bind with CD40 on monocytes in circulation to form platelet-leukocyte aggregates to promote inflammation through cytokine production. When platelets are activated, CD40L can be cleaved from the platelet surface to generate a soluble CD40L (sCD40L) molecule, which can also be released by immune cells such as T cells.

## 5. sCD40L and atherosclerosis

The exact role of inflammation in the development of atherosclerosis in different arterial regions remains not fully clear. Studies have shown that only cellular surface CD40L on endothelial cells can induce the expression of proinflammatory cytokines and adhesion molecules,^[73]^ while sCD40L has not been found to play a significant role in previous researches. However, sCD40L has been identified as a prothrombotic factor that stabilizes arterial thrombi through a β3-integrin-dependent mechanism. Elevated levels of sCD40L have also been observed in stable atherosclerosis in the carotid and coronary arteries.^[74]^ Therefore, sCD40L has been suggested as a predictive biomarker for primary and recurrent cardiovascular events, such as myocardial infarction and stroke.^[48,75,76]^ In recent studies, sCD40L has been shown to affect increasing lipid deposition and foam cell formation. Additionally, sCD40L increases cholesterol efflux and activates NF-κB in macrophages.^[77]^

In conclusion, sCD40L may serve as a promising noninvasive tool for refining the stratification of the systemic atherosclerotic burden or a predictive marker for cardiovascular events.^[78]^ But there were some recent short and long-term researches that failed to show the association of sCD40L with cardiovascular outcome and all-cause mortality. In the Ludwigshafen Risk and Cardiovascular Health study, sCD40L has been found to be uncorrelated with cardiovascular and all-cause mortality in this large cohort, and only in selected patient subgroups raised levels of sCD40L associated with short-term mortality.^[79]^ Therefore, the application of sCD40L as a predictor of atherosclerosis or cardiovascular events is still controversial now.

## 6. Polymorphisms of CD40 and CD40L in atherosclerosis

As tools for understanding genetic variation in diseases, sequence variations can be used for gene mapping and performance of functional studies. In recent years, single nucleotide polymorphisms (SNPs) have served as a hot topic in genetic research related to the association between functional molecules, genes, and diseases, including those related to immune regulation and vascular inflammation.^[80]^ To date, most research has primarily focused on the relationship between CD40 SNPs and atherosclerosis, while there have been limited investigations into CD40L polymorphisms. The CD40 gene contains several SNPs, including rs1883832, rs1535045, rs3765459, rs4810485, rs3092952, and rs3092920. However, only rs1883832 has been extensively studied for its correlation with atherosclerosis, yielding significant results. Specifically, the rs1883832 C/T polymorphism located within the Kozak sequence of the 5’-untranslated region of the CD40 gene has been linked to CD40 protein expression and an increased risk of developing atherosclerotic diseases. The recent study found that the C allele of CD40 rs1883832 triggers an inflammatory endothelial cells phenotype, which is compensated by enhanced CD40 shedding to neutralize excess CD40 ligand. And the homozygosity of the C allele is responsible for genetic susceptibility to atherosclerosis and its complications.^[81]^ The meta-analysis indicated that the CD40 gene had a dominant effect on atherosclerosis, and C allele rs1883832 was positively correlated with susceptibility to atherosclerosis in several Chinese population studies.^[82]^ Due to the limitations of meta-analysis, further fine-mapping and functional analysis of the CD40 gene region, as well as summarizing case-control studies in different populations and races,^[83–85]^ will be required in the future to identify causal variants and elucidate detailed mechanisms of CD40 polymorphisms in atherosclerosis.

## 7. Opportunities of CD40-CD40L in atherosclerosis treatment

Atherosclerosis is a vascular inflammatory process closely related to immune responses, and its initiation and progression involve the participation of second signal co-stimulatory molecules. Therefore, research on the association between arteriosclerosis and members of the co-stimulatory molecules has become a hot topic in recent studies. As a pair of important co-stimulatory dyad, inhibition of CD40-CD40L interactions has been proven effective in atherosclerosis initiation, prevention, regression, and plaque stabilization in a laboratory setting. However, prolonged interruption of this interaction can lead to severe immune suppression and other adverse effects.^[86]^ Therefore, it may be beneficial to target specific cell types or downstream components of the CD40 signal pathway cascade in atherosclerosis therapy.

**Figure 1.**
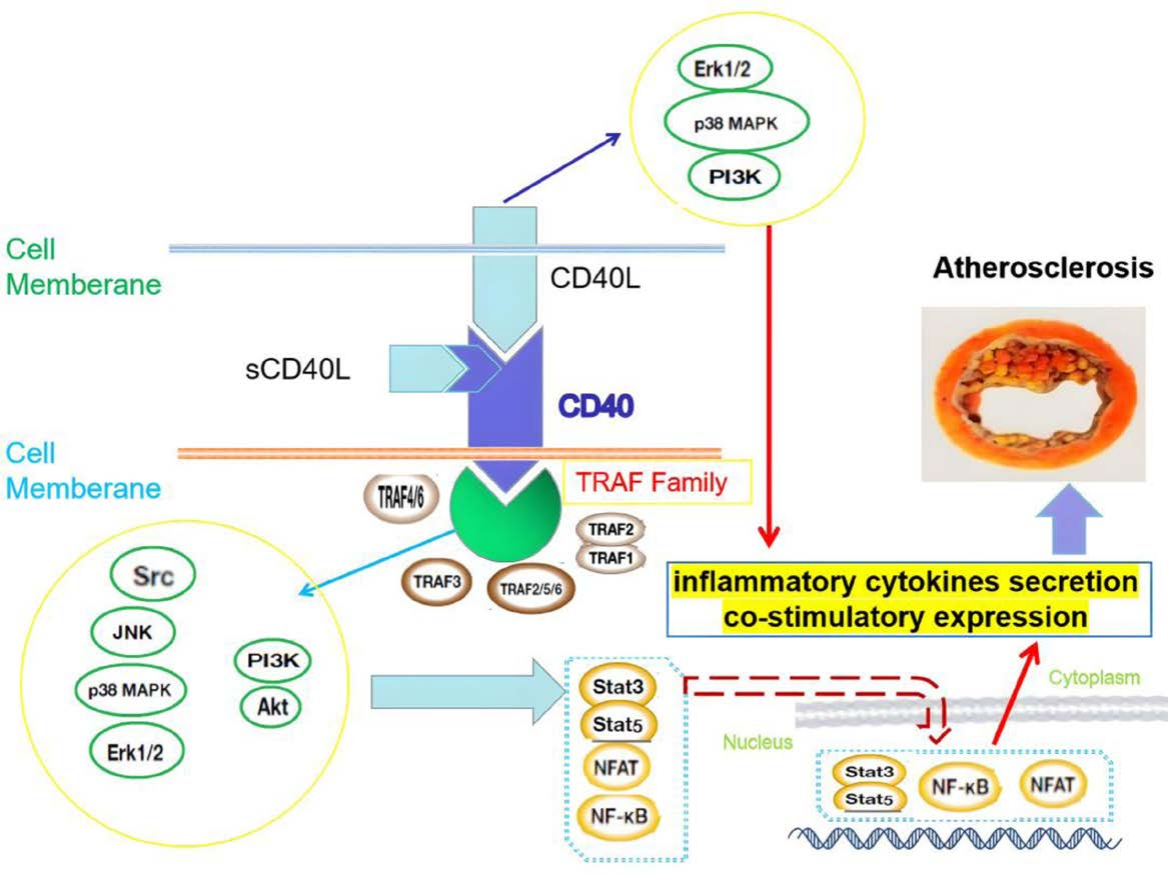
The role of CD40-CD40L pathway in inflammation leading to atherosclerosis.

According to Table 1, the CD40-CD40L signal system is cell type-specific and even varies within 1 cell type. Therefore, targeting different cell type-specific signal transduction pathways within the cell types present in atherosclerotic plaques could provide more potential treatment targets for atherosclerosis (Fig. 1). Additionally, genetic variability on CD40/CD40L genes appears to play a more significant role in atherosclerosis, such as through miRNA silencing of signaling pathways or SNPs affecting function regulation.^[87,88]^

**Table 1:** Potential functional targets of CD40-CD40L in atherosclerosis.

	Region or cell types	Phenotype characteristics	Potentially relevant stimulating elements	References no.
CD40	Monocytes/macrophages	Regulation of CD40L overexpression, affecting thrombosis	Signaling and transcription factors (TRAM, MAPK, STAT5, NF-κB) Secreting cytokines (IL-12, MMPs, MIP-1, MCP-1, IL-8, TNF-α, IFN-γ)	^[44,60]^
	Adipocytes, endothelial cells	Promotion and formation of a stable plaque phenotype	Leukocyte adhesion, inflammatory cell migration	^[51,52]^
	Dendritic cells	Attenuated atherosclerosis	Lipid uptaking	^[7,56]^
	Antigen-presenting cells	CD40-TRAF2/3/5 interaction	Inhibiting interaction between CD40 and TRAF6	^[61,62]^
	Endothelial cells	Determining the atherosclerotic susceptibility sites	Cytokines (ICAM-1, MCP-1, IL-6, IL-8, MMPs)	^[52,70–72]^
	SNP rs1883832	Triggering inflammatory phenotype of endothelial cells, associated with genetic susceptibility to atherosclerosis	C allele of CD40 in rs1883832	^[81,82]^
CD40L	Vascular ECs and SMCs in atherosclerotic plaque	Regulation of collagen, lipid, and inflammation Significant reduction in plaque area by anti-CD40L	Cytokines (IL-1, IL-6, IL-12, IFN-γ, TNF-α, IL-8, MIP-1α) Adhesion molecules (E-selectin, VACM-1, ICAM-1) Src family kinase pathway	^[33,49,50,64,65,67]^
	T cells	Regulation of thrombosis	Cytokines (IL-1β)	^[60]^
	Platelets	Triggering inflammatory responses and forming PLA	Cytokine production (MCP-1, IL-6, IL-8, MMPs)	^[70,71]^
sCD40L	Neutrophils	Stimulating more platelets	ROS	^[57,58]^
	Platelets	Stabilizing arterial thrombosis, increasing lipid deposition, and cholesterol efflux	β3-integrin, NF-κB	^[75–78]^

ECs = vascular endothelial cells, MMPs = matrix metalloproteinases, ROS = reactive oxygen species, SMCs = smooth muscle cells.

Lipid profiling is a targeted metabolomic platform that allows comprehensive analysis of lipid types. Various lipids are involved in the occurrence, development, diagnosis, and even treatment of atherosclerosis. The approaches based on synthetic high-density lipoproteins (HDL) administration have been proposed as significant immunomodulation and vascular protection by reducing inflammation and endothelial dysfunction.^[89,90]^ Research has shown that the level of HDL is correlated with the expression of sCD40L, and both can affect atherosclerosis.^[91]^ Therefore, the combined therapeutic target of CD40/CD40L and HDL in the lipid profile may be one of the future exploration directions for atherosclerosis.

For the CD40-CD40L dyad, continuous research in recent years has found that membrane-type or secreted CD40/CD40L is correlated with atherosclerosis, and may serve as a potential biomarker target serve as predictor, diagnose, or therapy.^[92–94]^ Several strategies have been developed to target CD40-CD40L signaling for atherosclerosis treatment. One approach is to inhibit the interaction between CD40L and CD40 using antibodies or antagonists. These agents can block the downstream signaling pathways that promote inflammation and atherosclerosis, leading to the reduction of plaque formation and stabilization of plaques. Another approach is to target CD40-CD40L signaling leading to the regulation of lipid metabolism and reducing the levels of proinflammatory cytokines. In the near future, there will be alternative approaches that are based on CD40-CD40L. As a result, the prospects for cell- or signal-specific treatment of the CD40-CD40L interaction in atherosclerosis are promising and will require long-term investigation.

## Author contributions

**Conceptualization:** Simeng Tian, Yufei Wang, Zhenkun Fu.

**Investigation:** Simeng Tian, Yufei Wang, Jie Wan.

**Writing—original draft:** Simeng Tian, Zhenkun Fu.

**Writing—review & editing:** Simeng Tian, Mao Yang, Zhenkun Fu.

**Funding acquisition:** Mao Yang.

**Supervision:** Mao Yang, Zhenkun Fu.
